# Did I Pick the Right Colony? Pitfalls in the Study of Regulation of the Phase Variable Antigen 43 Adhesin

**DOI:** 10.1371/journal.pone.0073568

**Published:** 2013-09-05

**Authors:** Ashwini Chauhan, Chizuko Sakamoto, Jean-Marc Ghigo, Christophe Beloin

**Affiliations:** 1 Institut Pasteur, Unité de Génétique des Biofilms, Département de Microbiologie, Paris, France; 2 Université Paris Diderot, Sorbonne Paris Cité, cellule Pasteur, Paris, France; Niels Bohr Institute, Denmark

## Abstract

Ag43 is an abundant outer membrane autotransporter adhesin present in most commensal and pathogenic *Escherichia coli*. Expression of the *agn43* gene is characterized by a regulated reversible switch or phase variation between the *agn43* ON and *agn43* OFF states. Although the *agn43* regulatory switch leads to a heterogeneous population of ON and OFF bacteria, studies of Ag43 seldom consider potential biases associated with phase variation. We monitored *agn43* ON/OFF phase-variation status genetically and phenotypically and we show that the use of populations with random *agn43* ON or OFF status could result in misleading conclusions about Ag43 function or regulation. In particular, we demonstrate that Lrp and MqsR, previously identified as *agn43* regulators, do not regulate *agn43* expression or ON/OFF switch frequency. We also show that biofilm formation in dynamic flow conditions does not influence *agn43* ON/OFF switching but physically selects aggregating *agn43* ON cells. This indicates that misinterpretation is possible when studying gene expression within biofilms. Finally, we provide evidence that ignoring the initial *agn43* ON/OFF status of the *E. coli* populations studied is likely to bias analyses of phenotypes associated with other *E. coli* adhesins. This study therefore emphasizes the importance of monitoring Ag43 phase variation and indicates that caution is required when interpreting experiments using strains that are neither deleted for *agn43* nor carefully assessed for *agn43* ON/OFF status.

## Introduction

Colonization of diverse environments by *E. coli* requires high adaptation abilities and a variety of colonization factors ensuring successful attachment to various surfaces. Recent post-genomic studies have demonstrated that *E. coli* indeed possesses a very large arsenal of adhesins with different specificities [1]–[11]. Two major families of adhesins have been identified in *E. coli*: adhesins carried by chaperone-usher fimbriae that generally recognize glycosylated proteins or lipids [12]–[14] and type V secretory autotransporter adhesins, recognizing specific receptors or self-associating, and implicated in bacterium-to-bacterium interactions [15]–[17]. Prototypical members of this family of self-associating autotransporters (SAATs) are AidA, an adhesin initially characterized in an *E. coli* O126:H27 strain isolated from a pediatric patient with diarrhea [18], TibA, first found in the ETEC O78:H11 strain H10407 [19], and the Antigen 43 adhesin (Ag43) one of the most abundant outer membrane proteins in *E. coli* [20], [21].

The gene coding for Ag43 is present in nearly all commensal and pathogenic *E. coli* and some isolates carry multiple copies of *agn43* alleles on pathogenicity islands [15], [22]. Whereas eukaryotic receptors specific for AidA and TibA have been identified, the only identified function of most Ag43 variants is the ability to promote bacterial autoaggregation and biofilm formation *in vitro*. *In vivo*, the Ag43 variant Ag43a was found to be involved in long-term persistence of uropathogenic CFT073 within mouse bladder [10]. Consistently, human epidemiological studies have associated the *agn43a* allele with UPEC persistence in bladder and recurrent infections [23].

**Figure 1:**
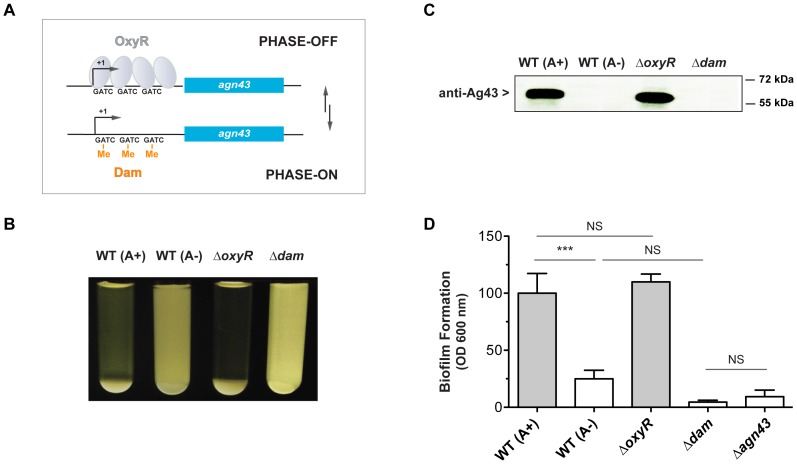
The natural *agn43* expression state (ON or OFF) strongly influences *E. coli* community behavior. **A.** Schematic representation of the *agn43* phase variation mechanism (not to scale): if OxyR binds to the *agn43* promoter, it impedes *agn43* transcription (PHASE OFF); however, if Dam methylates GATC sites at the OxyR binding site, *agn43* can be expressed (PHASE ON). This mechanism is heritable but reversible upon replication. **B.** Aggregating and non-aggregating clones from an isogenic wild-type TG strain. Pictures of stationary phase cultures were taken after 6 h settling on the bench. WT (A+): wild-type aggregating culture, WT (A−): wild-type non-aggregating culture, Δ*oxyR*: *agn43* locked-ON strain, Δ*dam*: *agn43* locked-OFF strain. **C.** Immunodetection of Ag43 in aggregating and non-aggregating clones. WT (A+): wild-type aggregating culture, WT (A−): wild-type non-aggregating culture, Δ*oxyR*: *agn43* locked-ON strain, Δ*dam*: *agn43* locked-OFF strain. **D.** Biofilm forming ability of an aggregating clone and a non-aggregating clone. Biofilms were formed in microfermentors for 24 h; quantitative analysis involved measuring the optical density of the resuspended biofilm. WT (A+): wild-type aggregating culture, WT (A−): wild-type non-aggregating culture, Δ*oxyR agn43* locked-ON strain, Δ*dam*: *agn43* locked-OFF strain, Δ*agn43*: deletion mutant of *agn43*. ***: p<0.0001. NS: not significant.

Remarkably, expression of *agn43* is phase variable and is characterized by ON and OFF states and switching rates of about 10^−3^ per cell per generation. This phase variable expression is due to the concerted action of a repressor, the oxidative stress regulator OxyR, and of an activator, the Dam methylase that methylates GATC sites in the regulatory region of *agn43* and overlaps with the OxyR binding site [24]–[28] (**Fig. 1A**). Most studies of the functions of Ag43 have been performed using strains overproducing Ag43 or containing mutations locking its expression in either the ON or OFF state therefore ignoring its natural phase variation. Any wild-type *E. coli* population is likely to be composed of a mixture of Ag43 ON and OFF bacteria, and the characterization of *agn43* regulators or studies of *agn43* expression using DNA arrays or RT-PCR experiments can be misleading due to absence of information about the Ag43 ON/OFF state of the bacterial populations tested (see results for *agn43*/*flu* regulation in GenExdb database - http://genexpdb.ou.edu/main) [29]–[36]. Indeed, van der Woude and Henderson suggested that differential expression observed in global expression analysis for genes subject to phase variation may be due to differences in the distribution (possibly random) of the ON/OFF cell ratio between bacterial populations rather than to genuine, robust regulatory differences [22].

In this study, we reinvestigated *agn43* regulation using a genetic approach allowing the *agn43* ON/OFF phase-variation status to be monitored while keeping a functional *agn43* gene. We confirmed that the *agn43* ON/OFF status strongly influences *E. coli* autoaggregation and biofilm formation, and demonstrate that biofilm formation leads to a physical selection of Ag43 ON bacteria therefore potentially biasing expression studies performed in biofilm condition. We show that ignoring the *agn43* ON/OFF status can introduce a substantial bias into phenotypic analyses of unrelated *E. coli* adhesins. Finally, the genetic tools developed in this study enabled us to show that Lrp and MqsR, previously identified as *agn43* regulators, do not regulate *agn43* expression or ON/OFF switching frequency [30], [33], [34]. Our study, therefore, illustrates the necessity of monitoring Ag43 phase variation and taking this phenomenon into account when studying bacterial adhesion and biofilm formation by *E. coli*.

## Materials and Methods

### Bacterial strains and growth conditions

Bacterial strains and plasmids used in this study are described in **Table 1**. All experiments were performed in 0.4% glucose M63B1 minimal medium (M63B1_Gluc_) or in Lysogeny Broth (LB) medium [37] at 37°C unless specified otherwise. Antibiotics were added as required to the following final concentrations: kanamycin (Km), 50 µg.mL^−1^; chloramphenicol (Cm), 25 µg.mL^−1^; zeocin (Zeo), 25 µg.mL^−1^.

**Table 1:** Strains and plasmids used in this study.

Strains	Relevant characteristics	Source or Reference
TG	TG1 cured of the F plasmid, proline auxotroph	[46]
MG1655 Δ*oxyR::Km*	MG1655 deleted for *oxyR*, Km^R^	[8]
MG1655 Δ*oxyR::Cm*	Deletion of *oxyR* in MG1655, Cm^R^	[53]
TG Δ*oxyR*	P1*vir* transduction into TG of Δ*oxyR* from MG1655 Δ*oxyR::Km*, Km^R^	This study
CSH50 Δ*dam*	CSH50 deleted for *dam*, Km^R^	[27]
TG Δ*dam*	P1*vir* transduction into TG of Δ*dam* from CSH50 Δ*dam*, Km^R^	This study
MG1655 Δ*agn43*	MG1655 deleted for *agn43*, Cm^R^	[8]
TG Δ*agn43*	P1*vir* transduction into TG of Δ*agn43* from MG1655 *Δagn43*, Cm^R^	This study
MG1655*lacZ*-*zeo*	MG1655 with the zeocin resistance gene introduced after the *lacZ* gene, Zeo^R^	[6]
TG *agn43-lacZ*	Insertion of *lacZ* reporter downstream from *agn43* by λ-red recombination, followed by P1*vir* transduction of *agn43-lacZ* in a TG background, Zeo^R^	This study
TG *Δagn43::lacZ*	Deletion of Δ*agn43*, replaced by *lacZ* reporter by λ-red recombination followed by P1*vir* transduction of *Δagn43::lacZ* in a TG background, Zeo^R^	This study
TG *agn43-lacZ ΔoxyR*	P1*vir* transduction into TG *agn43-lacZ* of Δ*oxyR* from MG1655 *ΔoxyR::Km*, Zeo^R^, Km^R^	This study
TG *agn43-lacZ* Δ*dam*	P1*vir* transduction into TG *agn43-lacZ* of Δ*dam* from CSH50 *Δdam*, Zeo^R^, Km^R^	This study
TG *Δagn43::lacZ* Δ*oxyR*	P1*vir* transduction into TG *Δagn43::lacZ* of Δ*oxyR* from MG1655 Δ*oxyR::Km*, Zeo^R^, Km^R^	This study
MG1655 PcL*-yfaL*	Insertion of the constitutive λp_R_ promoter (*Km*PcL casette) in front of *yfaL* and subsequent P1*vir* transduction in a MG1655 background, Km^R^	This study
TG *agn43-lacZ* PcL*-yfaL*	P1*vir* transduction into TG *agn43-lacZ* of PcL-*yfaL* from MG1655 PcL*-yfaL*, Km^R^, Zeo^R^	This study,
TG *Δagn43::lacZ* PcL*-yfaL*	P1*vir* transduction into TG *Δagn43::lacZ* of PcL-*yfaL* from MG1655 PcL-*yfaL*, Km^R^, Zeo^R^	This study
TG *agn43-lacZ ΔoxyR* PcL*-yfaL*	P1*vir* transduction into TG *agn43-lacZ* PcL-*yfaL* of Δ*oxyR* from MG1655 Δ*oxyR::Cm*, Km^R^, Zeo^R^, Cm^R^	This study
TG *Δagn43::lacZ* Δ*oxyR* PcL-*yfaL*	P1*vir* transduction into TG Δ*agn43::lacZ* PcL-*yfaL* of Δ*oxyR* from MG1655 Δ*oxyR::Cm*, Km^R^, Zeo^R^, Cm^R^	This study
JW0872	BW25113Δ*lrp::Km*FRT	[38]
TG *agn43-lacZ* -Δ*lrp*	P1*vir* transduction into TG *agn43-lacZ* of Δ*lrp::Km*FRT from JW0872	This study
JW2990	BW25113 Δ*mqsR::Km*FRT	[38]
TG *agn43-lacZ* Δ*mqsR*	P1*vir* transduction into TG *agn43-lacZ* of Δ*mqsR::Km*FRT from JW2990	This study

Plasmids	Relevant characteristics	Source or Reference
pKOBEG	pSC101 ts (replicates at 30°C), *araC*, arabinose-inducible λredγβα operon, Cm^R^	[39]

The *E. coli* strains used in this study were constructed by P1*vir* phage transduction from various strains including mutants from the Keio collection [38], or by using the λ-red linear DNA gene inactivation method [39], [40]. For construction of *lacZ* fusions we used the strain MG1655*lacZ-zeo* where the gene encoding zeocin resistance was placed after the *lacZ* gene on its native location [6]. The *E. coli* K-12 TG strain is a TG1 strain derivative commonly used in biofilm studies which has been cured of the F plasmid. TG possesses only one allele of *agn43*, located at the same chromosomal position as other K-12 laboratory strains. We constructed strain TG *agn43*-*lacZ* by introducing the *lacZ* gene with its own ribosome binding site (rbs) and the zeocin resistance gene after the stop codon of *agn43*; and strain TG Δ*agn43*::*lacZ-zeo* by replacing *agn43* (ATG to STOP codons) by *lacZ-zeo* keeping the rbs of *agn43*. The constitutive expression of *yfaL* was obtained by introducing, upstream from the ATG of *yfaL*, the *kmPcLrbs* cassette containing the constitutive λp_R_ promoter [41]. Primers used in this study are listed in **Table 2**. All constructions were confirmed by PCR and/or sequence analysis.

**Table 2:** Primers used in this study.

Primers	Sequence (5′ to 3′)	Target region/gene
end-agn43.lacZzeo.L-5	agggtataacggtcaggccacactgaatgtgaccttctgaatttcacacaggaaacagct	insertion *lacZ* after *agn43*
end-agn43.lacZzeo.L-3	ccggtcatgatgaccgggaccacagagaggcgatggttcttcagtcctgctcctcggccac	insertion *lacZ* after *agn43*
agn43.ext-3	atcagtgacggtgaaatat	*agn43* verification
end-agn43.ext-5	aagcgtcatcggacaataac	*agn43* verification
lacZ.ATG+100-3	gggggatgtgctgcaaggcgattaag	*lacZ* cassette-gene junction verification
zeo.verif-5	caggaccaggtggtgccggacaacaccc	*lacZ* cassette-gene junction verification
agn43.lacZzeo.L-5	taccggcttttttattcaccctcaatctaaggaaaagctgatgaccatgattacggattc	replacement *agn43* by *lacZ*
agn43.lacZzeo.L-3	tcatgatgaccgggaccacagagaggcgatggttctgtcagtcctgctcctcggccac	replacement *agn43* by *lacZ*
agn43.ext-5	atacgctggtcagtgcgctc	*agn43* deletion verification
MqsR-500-5	gacgaccctgccaccaccgca	*mqsR* verification
MqsR-500-3	caacaacaatacgcctgtggcat	*mqsR* verification
Lrp-500-5	gagatccccatagttgttgg	*lrp* verification
Lrp-500-3	agaccacaggaggtaaggat	*lrp* verification
yfaL.PcLKmrbs.L-3	gtaaagataaatactccttgcgtagaaagataatccgcatgcggtacctttctcctctttaatg	Insertion of KmPcLrbs in front of *yfaL*
yfaL.PcLKmrbs.L-5	ttccatatcgtataatgcgattaaatacgccgtcttatagttcgctcaagttagtaattctcac	Insertion of KmPcLrbs in front of *yfaL*
yfaL.A1.500-5	ggtcagacaaggtgtccggg	*yfaL*
yfaL.ext-5	cataactttgtggataactcagg	*yfaL*
yfaL.B1. PcLKmrbs-500-3	cgttagtgacacgtaaatcg	*yfaL*
yfaL. PcLKmrbs.ext-3	cattattaatggtataaattg	*yfaL*
yfaL.ATG+100-3	caactcgctttgacatcatatc	*yfaL*
PcL-km.verif-5	cagagcagccgattgtctgttg	cassette-gene junction verification
PcL-km.verif-3	cttcctcgtgctttacggtatcg	cassette-gene junction verification

### Switch frequencies

The Ag43 switch frequencies were calculated as described previously [27], [42]. Briefly, five blue or five white colonies were suspended in 1 mL LB medium and dilutions were plated on LB agar plates supplemented with 100 µg.mL^−1^ of 5-bromo-4-chloro-3-indolyl- β-D-galactopyranoside (X-gal). The plates were incubated overnight at 37°C. The blue and white colonies were counted and used to calculate the switch frequencies. Both total counts of viable cells (N) and the number of colonies that switched from the phenotype of the original inoculum (M) were determined. Based on the assumption that predominantly phase-ON and phase-OFF colonies are derived from phase-ON and phase-OFF cells, respectively, the following equation was used to calculate the frequency of phase switching: switching frequency (per cell per generation) = 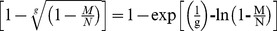 where g is the number of generations of growth and is calculated as g = (logN/log2).

### Autoaggregation Assay

Aggregation assays were performed as described in [8]. Isolated blue or white colonies were picked from LB/X-gal plates, and individual colonies were used to inoculate 5 mL LB medium and grown overnight (16–18 h). The optical density of the culture at 600 nm (OD_600_) was adjusted to 3.0 by dilution with nutrient-exhausted LB medium (supernatant obtained from respective overnight grown cultures after centrifugation), and 3 mL of each adjusted culture was transferred to 5 mL hemolysis tubes. These tubes were incubated without agitation at room temperature. The OD_600_ of the upper part of each standing tube culture was determined every hour for 8 h.

### Biofilm formation assay in micro-titer plates

Biofilm formation was assayed by determining the ability of cells to adhere to the wells of 96-well polyvinyl chloride (PVC) micro-titer plates [43], [44]. An overnight culture in M63B1_Gluc_ supplemented with 0.4 mg.mL^−1^ proline was inoculated at a 1/100 dilution (1 µL inoculum in 100 µL medium per well) in the same medium and the plates were incubated at 37°C. After 24 h of growth, wells were rinsed with H_2_O, and 125 µL of a 1% solution of crystal violet was added to each well. The plates were incubated at room temperature for 15 min and rinsed. Crystal violet was solubilized by addition of 200 µL of ethanol-acetone (80∶20), and the OD_570_ was determined. The results are averages for four replicate wells in three independent experiments.

### Biofilm formation assay in microfermentors

All experiments were performed in triplicate in M63B1_Gluc_ medium supplemented with 0.4 mg.mL^−1^ proline at 37°C. Sixty-milliliter microfermentors containing a removable glass slide were configured as continuous-flow culture bioreactors with a flow rate of 40 mL.h^−1^ [45], [46]. Bacterial inocula equivalent to an OD_600_ of 1 from overnight precultures grown in M63B1_Gluc_ medium supplemented with 0.4 mg.mL^−1^ proline and appropriate antibiotics were used to inoculate the microfermentors; the cultures were then cultivated for 24 h and 48 h. Images of each removable glass slide were captured at the end of the incubation period. After 24 h or 48 h of growth the biofilm on the slide was resuspended in 10 mL of M63B1 medium and the OD_600_ of the suspension was determined. The resuspended biofilms were also used to determine percentages of cells in the ON and OFF states, by immunofluorescence in the case of the TG *agn43*-*lacZ* strain or by plating on LB-Xgal agar plates for the TG Δ*agn43::lacZ-zeo* strain.

### Immunofluorescence

Immunofluorescence microscopy analysis was performed as previously described [41]. Briefly, strains were cultured overnight at 37°C in LB medium with the appropriate antibiotics. Overnight cultures were diluted to OD_600_ 1 in 1× PBS and aliquots were loaded onto 0.1% poly-L-lysine-treated immunofluorescence microscope slides. A 1∶1,000 dilution of primary polyclonal rabbit anti-serum raised against the α-domain of Ag43 was used to label Antigen 43 (antibodies given by P. Owen). A 1∶300 dilution of a secondary polyclonal goat anti-rabbit serum coupled to Alexa488 (Molecular Probes-Invitrogen) was used to reveal bound antibody and 10 µg.mL^−1^ 4′,6-diamidino-2-phenylindole (DAPI) was used to stain the bacterial DNA nucleoid. The slides were mounted with Mowiol 4088 (Calbiocem) and observed under an epifluorescence microscope with green fluorescent protein and DAPI filters.

### Ag43 immunodetection

For each culture, the equivalent of 0.2 OD_600_ units was analyzed by sodium dodecyl sulfate—10% polyacrylamide gel electrophoresis, followed by immunodetection of Ag43. Protein loading accuracy was verified using staining of membrane with Ponceau S. When necessary, the α-subunit of *E. coli* RNA polymerase (Neoclone biotech) was used as an internal control. A polyclonal rabbit antiserum raised against the α-domain of Ag43 was used at a dilution of 1∶10,000 for immunodetection and the antibody specific for the α-subunit of *E. coli* RNA polymerase was used at a dilution of 1∶15,000.

### RNA isolation and semi-quantitative RT-PCR

Bacterial strains were grown overnight in LB medium and their RNA was extracted using the RNeasy Protect Bacteria Mini-Kit (Qiagen). Extracted RNA was treated with RNase-free DNase, repurified and stored at −80°C. RNA at a concentration of 500 ng.µL^−1^ was used for cDNA synthesis by Superscript II (Invitrogen Life Technologies) and 150 ng random primers (mostly hexamers). The obtained cDNA was diluted 1/1, 1/10 and 1/100 and the transcripts for the *agn43* and *16S* were amplified (94°C 1 min, 60°C 1 min, 72°C 1 min, for 30 cycles) using Supermix and ExTaq polymerase (TaKaRa) with 10 mM of the appropriate primers (see **Table 2**). Non-reverse-transcribed RNA was used as a negative control to confirm the absence of contaminating genomic DNA.

### Statistical analysis

Results presented are means +/− standard deviation. Statistical differences were evaluated using one-way ANOVA (Tukey multiple comparison test) included in Graphpad Prism Version 5.0c. The treatment groups were considered significantly different if p-values were lower than 0.05.

## Results

### Ignoring Ag43 phase-variation status randomizes analyses of *E. coli* aggregation phenotypes

To determine to what extent the naturally occurring *agn43* phase variation in *E. coli* influences its community behavior, we streaked the wild-type *E. coli* strain K-12 TG on LB agar plates from a −80°C glycerol stock. One hundred isolated colonies were used to inoculate LB and grown overnight at 37°C. We tested the auto-aggregation properties of each individual overnight culture as a marker of Ag43 expression status. Only five of the 100 colonies displayed an auto-aggregation phenotype (A+) (**Fig. 1B**). Serial dilutions of one of these (A+) aggregating cultures was plated on LB agar plates, and 100 isolated colonies were used to inoculate liquid cultures to re-test their aggregation phenotype. This time, 75% percent of these cultures aggregated (A+) and 25% did not (A−). Immunodetection using Anti-Ag43 antibodies were used to test for Ag43 in one aggregating culture and one non-aggregating culture. This analysis showed that the presence of Ag43 correlated with the aggregation phenotype (**Fig. 1C**). Moreover, aggregating clones (A+), but not non-aggregating clones (A−), displayed strong biofilm forming ability in a continuous flow system (**Fig. 1D**). Despite the observed correlation between auto-aggregation and Ag43 production, the A+ and A− phenotypes obtain with this wild-type phase variable strain were less marked than those of control strains locked-ON (*oxyR*) or locked-OFF [5] for *agn43* expression (**Fig. 1B** **)**. We tested whether mixtures of Ag43+ and Ag43− bacteria, in various proportions, could determine the degree of aggregation of the corresponding culture: locked-OFF *dam* mutant bacteria were mixed with locked-ON *oxyR* mutant bacteria in various ratios. The degree of aggregation was directly proportional to the number of Ag43+ bacteria (see **Fig. S1**). These results demonstrate that streaking −80°C stocks of *E. coli* results in a heterogeneous population of colonies, some expressing and some not expressing Ag43; consequently, picking an ON or OFF colony at random strongly influences the outcome of analyses of bacterium-bacterium interactions.

### Monitoring the *agn43* expression state using an *agn43*-*lacZ* operon reporter fusion

**Figure 2:**
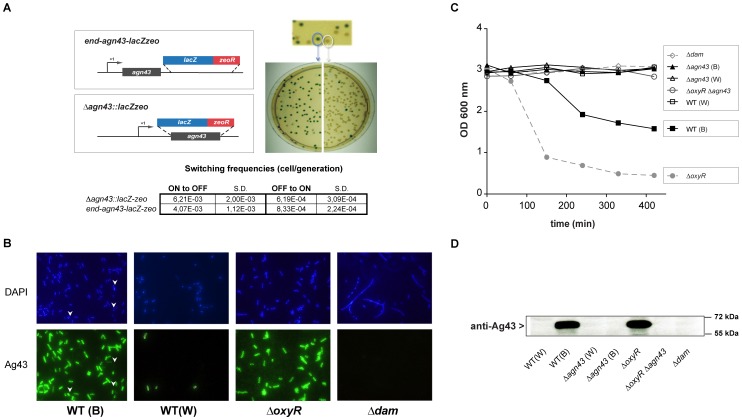
Construction and characterization of *agn43-lacZ* transcriptional fusions. **A.** Schematic representation of TG *agn43-lacZ* and TG *Δagn43::lacZ* fusions (not to scale). Blue or white colony plated on LB agar+X-gal plates: a blue colony gives rise to blues and whites and *vice versa*. Switching frequencies (ON or OFF cells/generation) of the transcriptional fusions were calculated as described in the materials and methods. S.D.: standard deviation. **B.** DAPI and immunofluorescence microscopy of a TG *agn43-lacZ* blue (ON) colony and a white (OFF) colony. Anti-Ag43 polyclonal antibody was used to detect surface-exposed Ag43. WT(B): wild-type ON colony with OFF cells pointed out by white arrow heads, WT(W): wild-type OFF colony, Δ*oxyR*: *agn43* locked-ON strain, Δ*dam*: *agn43* locked-OFF strain. **C.** Kinetics of aggregation of overnight cultures inoculated with TG *agn43-lacZ* (WT) and *Δagn43::lacZ* strains (*Δagn43*) blue or white colony. **D.** Immunodetection of Ag43 in TG *agn43-lacZ* and *Δagn43::lacZ* cultures started with either an ON or OFF colony, using an anti-Ag43 polyclonal antibody. In a TG *agn43-lacZ* background, WT(W): OFF colony, WT(B): ON colony, Δ*oxyR*: locked-ON strain, Δ*dam*: locked-OFF strain. In TG *Δagn43::lacZ* background, Δ*agn43* (W): OFF colony, Δ*agn43* (B): ON colony, Δ*oxyRΔagn43*: locked-ON strain.

To alleviate the uncertainty about the *agn43* ON/OFF expression status of an *E. coli* inoculum, we created a strain allowing direct distinction between colonies in the *agn43* ON and OFF states. We inserted the ß-galactosidase *lacZ* gene immediately downstream of the *agn43* coding sequence to generate an operon consisting of *agn43* and *lacZ* in *E. coli* TG. This construction at *agn43* chromosomal locus, allows the production of a functional Ag43 protein and is stable without any antibiotic selection pressure. The *E. coli agn43*-*lacZ* strain generated both blue (ON) and white (OFF) colonies on X-gal plates, and the switching frequency from ON to OFF was ≈5.10^−3^ cell/generation and from OFF to ON was ≈5.10^−4^ cell/generation (**Fig. 2A**). These frequencies are consistent with previous reports, and indicate that expression of the *agn43*-*lacZ* operon is subject to *agn43* phase variation [42]. Immunolocalization experiments confirmed the presence of Ag43 at the cell surface of most or few bacteria in blue and white colonies respectively (**Fig. 2B**): blue colonies were composed of 83+/−2% of ON bacteria whereas white colonies contained 96+/−1% of OFF bacteria. Consistently with results obtained with wild-type *E. coli*, a culture of a blue colony, which does not contain solely *agn43* ON bacteria, aggregated less (**Fig. 2C**) and produced slightly less Ag43 (**Fig. 2D**) than cultures originating from a *ΔoxyR* mutant (locked-ON). These results show that this *agn43*-*lacZ* strain faithfully reproduces both *agn43* phase variation and associated phenotypes.

### Physical selection, and not switch towards *agn43* ON state, results in higher Ag43+ populations in biofilms

Previous reports suggested that heterogeneity in Ag43-mediated cellular aggregation may constitute a selective bias in certain experimental situations [35]. We studied this possibility by monitoring the evolution of the *agn43* ON/OFF state during biofilm formation in a continuous flow system; in this system, population of Ag43+ cells may have an advantage, therefore introducing potential bias in gene expression analysis. We inoculated continuous flow biofilm microfermentors with bacterial populations grown either from an ON (blue) colony or an OFF (white) colony. In parallel, planktonic cultures were grown from the same inocula for 24 h and 48 h. We used Ag43 immunofluorescence to estimate proportion of ON and OFF cells in the initial inocula, and in 24 h and 48 h planktonic and biofilm populations.

**Figure 3:**
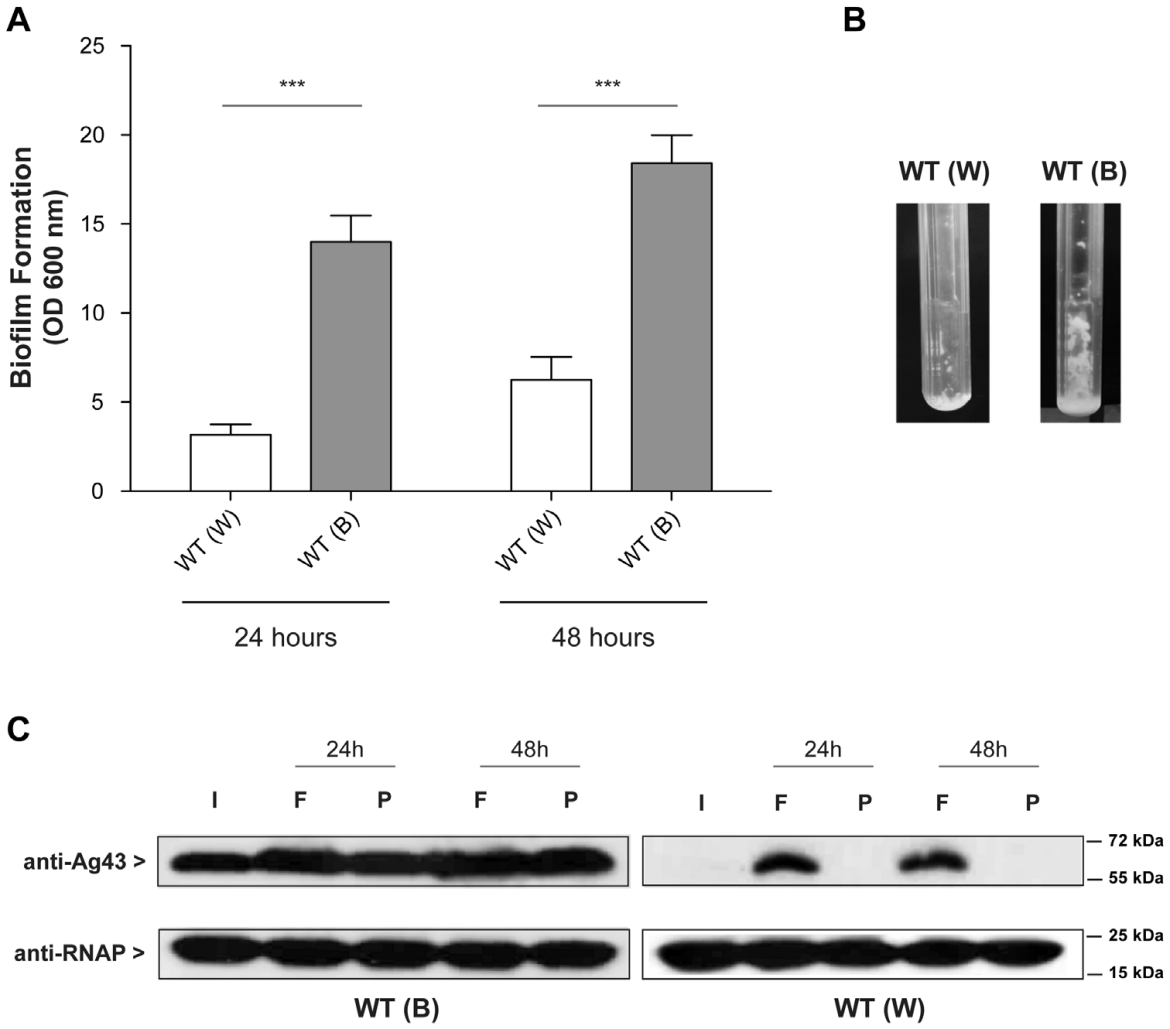
*In vitro* biofilm produced in continuous flow culture bioreactors selects for *agn43* ON cells. **A.** Biofilms of TG *agn43-lacZ* from an ON or an OFF colony in microfermentors. The biofilms were grown for 24 and 48 h; the biomass growing on the spatula was resuspended in 10 mL M63B1_Gluc_ and the optical density at 600 nm was measured. (B): ON colony, (W): OFF colony; **B.** Pictures of microfermentors after 48 h growth. ***: p<0.0001. **C.** Immunodetection of Ag43 in TG *agn43-lacZ* biofilm (F) or planktonic cultures (P) from ON (B) or OFF (W) colonies. I: inoculum, F: microfermentor biofilm, P: planktonic culture, the **α**-subunit of RNA polymerase (RNAP) was used as an internal control.

Biofilm formation in microfermentors was greater following inoculation with a culture originating from an ON colony than from an OFF colony (**Fig. 3A**); this was consistent with the capacity of Ag43 to promote bacterial aggregation. However, biofilm biomass increased between 24 h and 48 h independently of the initial *agn43* ON/OFF state of the inoculum (**Fig. 3B**). Moreover, the proportion of ON cells increased substantially over time within biofilms regardless of the initial *agn43* ON/OFF state, whereas it changed only moderately in planktonic cultures (**Table 3**). This was especially striking with *agn43* OFF cells inocula: ON cells made up only 2.3% of the initial population, but were 55% in biofilms after 48 h, and 7.3% in planktonic culture after 48 h. Immunoblot was used to detect Ag43 protein: it was more abundant in biofilms than in the corresponding planktonic cultures, where almost no change compared to inoculum was detected (**Fig. 3C**). Therefore the proportion of Ag43+ bacteria in biofilm increases, irrespective of the initial *agn43* ON/OFF state.

**Table 3:** Percentages of ON and OFF cells in 24/48 h-old biofilms or planktonic cultures of strain TG *agn43-lacZ*.

		OFF colony		ON colony	
		% OFF	% ON	% ON	% OFF
**Fermentor**	inoculum*	97.6	2.4	93.5	6.5
	24 h	73.1	26.9	96.5	3.5
	48 h	44.8	55.2	99.0	1.0
**Planktonic**	inoculum*	97.6	2.4	93.5	6.5
	24 h	93.7	6.3	82.8	17.2
	48 h	92.6	7.4	83.2	16.8

* ON or OFF colonies were used to grow the inocula. The same inoculum was used for fermentors and planktonic cultures.

This positive selection for *agn43* ON cells could result from a physical selection of Ag43+ cells or from an increased OFF to ON switching frequency during biofilm formation. To address this issue, we replaced *agn43* in its native chromosomal locus with the *lacZ* gene, thereby generating strain *E. coli* TG *Δagn43::lacZ*: in this strain, *agn43* promoter remains subject to phase variation but there is no production of Ag43 such that the strain is non-aggregating (**Fig. 2**). Because of the *agn43* deletion, the proportion of ON/OFF Ag43 cells during biofilm formation could not be evaluated by immunofluorescence and was determined by plating and counting blue and white colonies. Surprisingly, we did not observe any difference in the percentages of *agn43* ON/OFF cells between biofilm and planktonic *E. coli* TG *Δagn43::lacZ* populations (**Table 4**). These results demonstrate that the increased Ag43 expression in biofilms is due to physical selection of ON cells in the biofilm, rather than switching towards the *agn43* ON phase.

**Table 4:** Percentages of ON and OFF cells in 24/48 h-old biofilms or planktonic cultures of strain TG *Δagn43::lacZ*.

		OFF colony		ON colony	
		% OFF	% ON	% ON	% OFF
**Fermentor**	inoculum*	98.6	1.4	89.4	10.6
	48 h	94.3	5.7	81.7	18.3
**Planktonic**	inoculum*	98.6	1.4	89.4	10.6
	48 h	91.8	8.2	90.9	9.1

* ON or OFF colonies were used to grow the inocula. The same inoculum was used for fermentors and planktonic cultures.

### The Ag43 state biases phenotypic analysis of the function of *E. coli* adhesins

**Figure 4:**
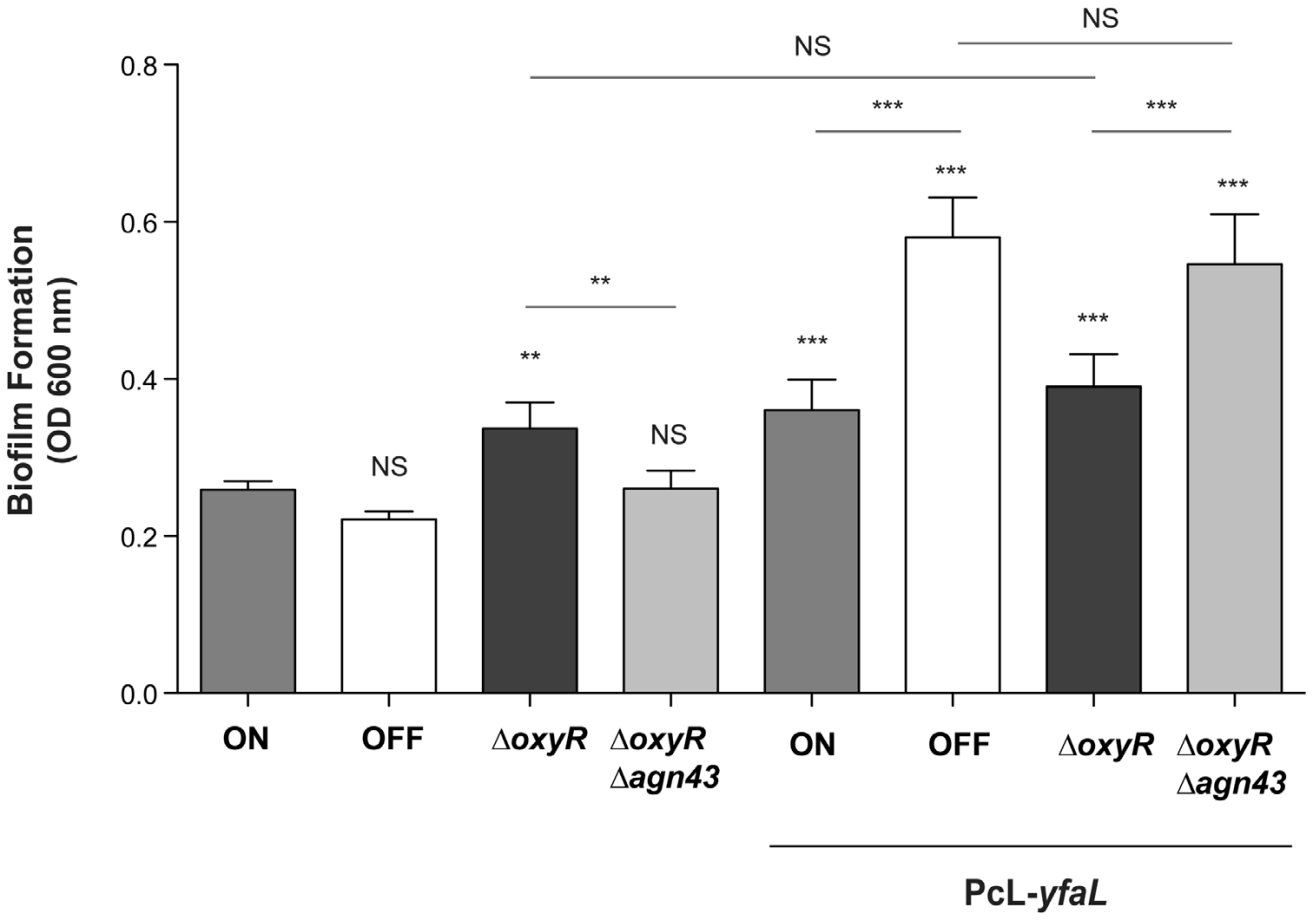
Ag43 interferes with YfaL-mediated biofilm formation. Biofilms were formed in 96-well micro-titer plates for 24 h; biofilm production was quantified by crystal violet staining as described in materials and methods. A TG *agn43-lacZ* background was used to monitor the ON or OFF state of colonies, ON: wild-type ON colony, OFF: wild-type OFF colony. PcL-*yfaL* strains constitutively expressing *yfaL*: *agn43* ON/OFF colonies, Δ*oxyR*: locked-ON strain, Δ*oxyR Δagn43*: Δ*agn43*::lacZ locked-ON strain. Unless specified statistical analyses were performed using the WT (ON) strain as a reference: NS: not significant, **: p<0.001, ***: p<0.0001.

Our results indicate that the outcome of adhesion and biofilm studies in *E. coli* depends largely on whether *E. coli* colonies originating from Ag43 ON or Ag43 OFF bacteria are used. This raises a question of whether the initial Ag43 ON or OFF state of *E. coli* cultures also biases the analysis of adhesion and biofilm phenotypes mediated by potential uncharacterized adhesins other than Ag43. We therefore investigated the role of Ag43 phase variation status on phenotypes mediated by the potential autotransporter adhesin YfaL, previously shown to increase *in vitro* biofilm formation by *E. coli* [8]. A genetic construction constitutively expressing *yfaL* (PcL-*yfaL*) was introduced into our *agn43-lacZ* reporter strain and we tested the ability of the resulting *E. coli agn43-lacZ* PcL-*yfaL* blue (ON) and white colonies (OFF) to form biofilm in the widely used micro-titer plate assay. This assay has been extensively used as a straightforward assay for evaluating bacterial adhesion properties in most studies related to biofilms. The constitutive expression of *yfaL* led to significantly more biofilm formation when the inoculum originated from an OFF than ON colony (**Fig. 4**). Also, *yfaL* expression from PcL-*yfaL* did not promote biofilm formation in an *oxyR* mutant, in which cells are 100% ON, whereas deletion of *agn43* in this *oxyR* context restored the enhancement of biofilm formation by YfaL (**Fig. 4**). This shows that Ag43-mediated aggregation can affect the outcome of biofilm or adhesion experiments; such analyses should always be performed in a genetic background where *agn43* status can be monitored or in a Δ*agn43* background.

### Despite previous reports, neither Lrp nor MqsR regulate *agn43*

**Figure 5:**
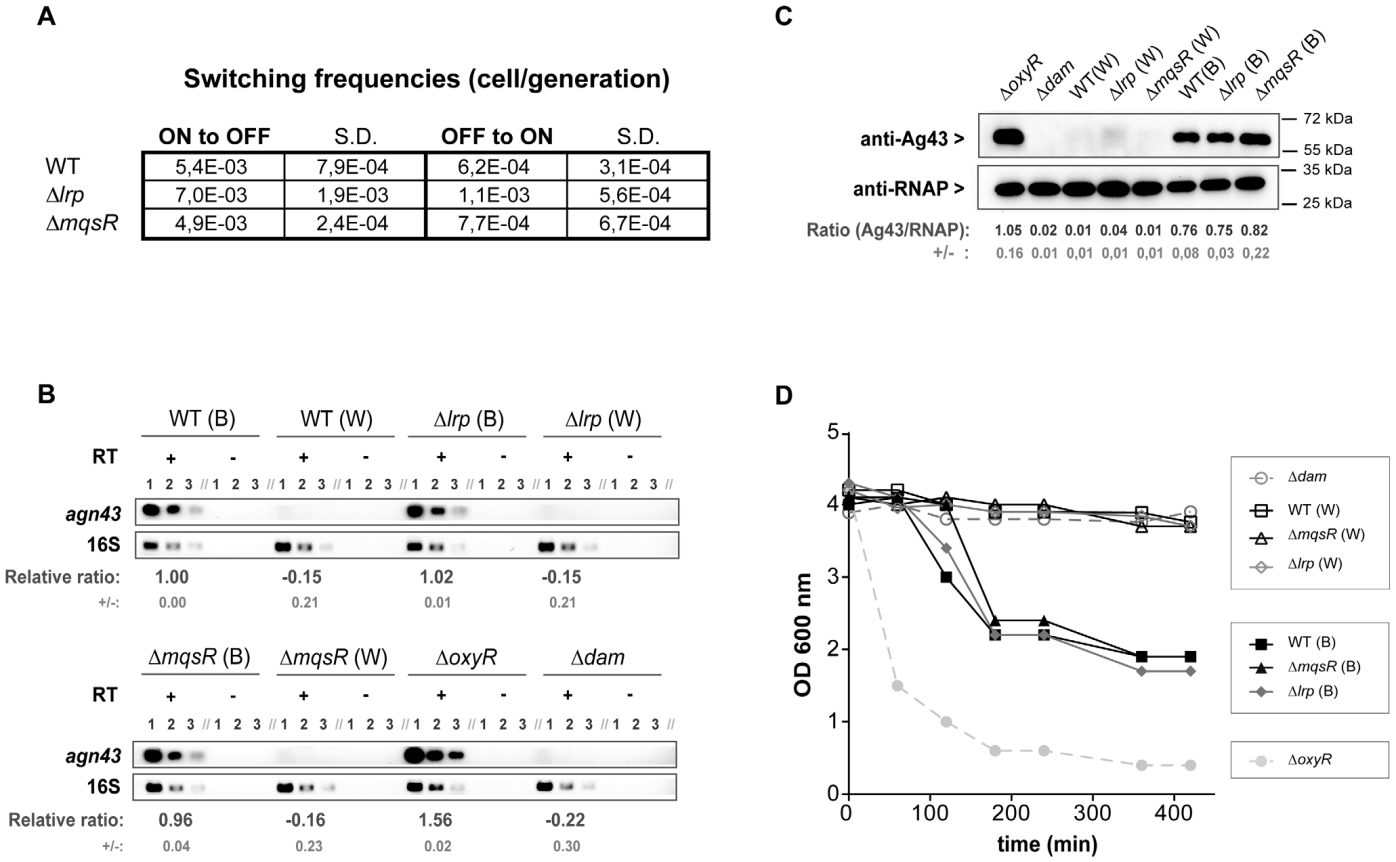
Lrp and MqsR do not regulate *agn43* expression. A. Switching frequencies of *lrp* and *mqsR* mutants assessed with an *agn43-lacZ* fusion. S.D.: standard deviation. B. Semi-quantitative RT-PCR analyses of *agn43* expression in wild-type (WT), Δ*lrp*, Δ*mqsR*, Δ*oxyR*, and Δ*dam agn43-lacZ* strains. Experiments were performed using RNA preparations that were not diluted (1), or diluted 1/10 (2) or 1/100 (3). (B): ON colony, (W): OFF colony, +/− RT: with or without reverse transcriptase polymerase. The 16S (*rrsh* gene) was used as an internal control. Relative ratio: average of *agn43*/16S band intensity ratio quantified using ImageJ, WT(B) used as reference; +/−: standard deviation. C. Immunodetection of Ag43 with anti-αAg43 polyclonal antibody in WT, Δ*lrp* and Δ*mqsR agn43-lacZ* strains. (B): ON colony, (W): OFF colony, the α-subunit of RNA polymerase (RNAP) was used as an internal control. Relative ratios (Ag43/RNAP): band intensity ratio quantified using ImageJ; +/−: standard deviation. D. Kinetics of aggregation of strains TG *agn43-lacZ*, TG *agn43-lacZ Δlrp* and TG *agn43-lacZ ΔmqsR*. (B): ON colony, (W): OFF colony, Δ*oxyR*: locked-ON strain, Δ*dam*: locked-OFF strain.

Ignoring the *agn43* expression status could also skew whole population analyses and lead to erroneous identification of regulators of *agn43* expression or ON/OFF switching frequency. To illustrate this point, we used the *E. coli agn43*-*lacZ* strain to study the roles of the previously identified *agn43* regulators Lrp and MqsR. These factors were identified as *agn43* regulators by transcriptomic approaches [30], [34]. It has been reported that deletion of the *lrp* and *mqsR* genes reduce *agn43* expression by almost 5-fold [34] and 17-fold [30], respectively. However, their deletions from TG *agn43*-*lacZ* had no effect on *agn43* switching frequencies (**Fig. 5A**) or on the amount of *agn43* transcripts as assessed by RT-PCR with blue and white colonies (**Fig. 5B**); Ag43 levels in the *lrp* and *mqsR* mutants were not different to that in wild-type (WT) cells (**Fig. 5C**), and these mutations did not modify the auto-aggregation properties of blue or white colonies (**Fig. 5D**).

Thus, neither Lrp nor MqsR regulate *agn43* expression or ON/OFF switching frequencies in our genetic background, evidence that ignoring the phase variation status of *agn43* in the studied population can result in major biases in whole-population *agn43* gene expression analyses.

## Discussion

There have been numerous studies of Ag43 phase variation, but few investigated how this unusual regulatory process interferes with *E. coli* adhesion to a surface. Here, we demonstrate that ignoring the *agn43* ON/OFF status can make studies of both the regulation and function of *agn43* extremely difficult to interpret.

We show that the simple and mundane act of picking a colony to start an *E. coli* culture is equivalent to a random choice of an *agn43* ON or OFF colony, unless an appropriate detection approach is used. As there is a higher probability of switching from ON to OFF than OFF to ON, the odds of working with an OFF colony for Ag43 are higher than those of working with an ON colony. This difference in switching explains why an OFF colony of the *E. coli* TG strain used in our study gave rise to a population of 95% of OFF and 5% of ON whereas an ON colony gave rise to a population of 75% of ON and 25% of OFF. The phenotypic consequences of this are such that the initial *agn43* phase variation status should be determined for all phenotypic analyses of *E. coli* adhesion and biofilm formation.

We developed a genetic tool for monitoring the Ag43 status in an *E. coli* background that is wild-type with respect to Ag43 production. Our *agn43-lacZ* fusion could be used to study *agn43* regulation and Ag43 function, taking Ag43 phase variation into account. This approach could also easily be applied to pathogenic *E. coli* and it would be possible to construct different reporter fusions if multiple *agn43* variants are expressed.

Using the *agn43-lacZ* reporter strain, we demonstrated a strong correlation between the ON or OFF colony status and aggregation phenotype. Nevertheless, ON state colonies aggregated less than *oxyR* mutant colonies locked in the ON state. Using various proportion of ON and OFF bacteria we showed that this is probably due to the proportion of ON bacteria being lower in an ON colony than in an *oxyR* colony, where 100% of the bacteria are in the ON state. Intriguingly, although wild-type aggregating clones aggregated less than an *oxyR* mutant, they formed as much biofilm as the *oxyR* mutant in continuous flow biofilm fermentors. Conversely, and albeit to a lesser extent, wild-type non-aggregating clones formed better biofilms than either *dam* or *agn43* mutants. This indicates either an imperfect direct correlation between Ag43-mediated auto-aggregation and biofilm formation, or a phenomenon of positive selection of Ag43+ bacteria during biofilm formation.

Our results are consistent with the second of these two possibilities; we found that, although planktonic and biofilm cultures displayed similar *agn43* switching frequencies, Ag43+ bacteria were positively selected in continuous flow biofilm fermentors. We therefore concluded that the high Ag43 level in biofilms is due to physical selection of ON cells rather than increased switching towards the *agn43* ON phase. Possibly Ag43− cells contribute less to biofilm formation than Ag43+ cells and are less well integrated and more easily washed out in experimental systems in which biofilms are formed in dynamic flow conditions. Our results constitute proof of principle in a specific situation in which Ag43+ cells can be enriched and become a potential source of bias in an analysis of Ag43-related function or *agn43* regulation. They are also consistent with the idea advanced by Tree *et al.* that the phenotypic heterogeneity generated by *agn43* phase variation could bias studies due to selective advantage for Ag43-mediated cellular aggregation [35]; the authors suggested that the deletion of the *cueO* gene, encoding a multicopper oxidase, derepressed the expression of *agn43* indirectly by a natural selection of ON cells in the population without affecting the level of *agn43* expression per ON cell.

The positive selection of Ag43+ cells that we observed in biofilm *in vitro* can presumably also occur *in vivo*, for example, in intracellular bacterial communities (IBCs) formed in bladder. The initial colonization of bladder epithelium, mediated by type 1 fimbriae, results in exfoliation of superficial cells, causing many bacteria to be shed in urine, but numerous bacteria remain attached to the urothelium [47]. This phenomenon may result in selective retention of Ag43+ cells: Ag43 may allow initial tighter adherence of bacteria followed by autoaggregation. Indeed, different variants of Ag43 have been shown to mediate adhesion to renal proximal tubular cells and kidney cell line [48] and a positive selection of Ag43+ cells would explain the strong expression of Ag43 described in intracellular biofilm communities (IBCs) formed by uropathogenic *E. coli* within murine bladder cells [49]. However, the fact that this strong expression was localized is also consistent with clonal expansion from a small number of original Ag43 ON cells rather than a positive selection for an Ag43+ population. These observations further illustrate the existence of such Ag43 phase variation mechanisms *in vivo*, and also show that more work is needed to elucidate the behavior of Ag43 ON and OFF cells *in vivo*.

Our study also suggests that global gene expression analysis is not appropriate for the analysis of Ag43 regulation. For instance, it has been suggested that the 14-fold increase in expression of *agn43* associated with the mutation of *cueO* (the gene for the periplasmic multicopper oxidase) is not a direct regulatory effect but the consequence of the selection of cells with ON phenotype within the population [35]. Here, we show that *lrp* and *mqsR* mutants, previously identified by transcriptomic methodology as potential *agn43* activators, did not affect either *agn43* expression or Ag43 function. Although we cannot exclude that the reported regulations could be strain specific as they were done in W3110 or MG1655 genetic backgrounds that do not markedly differ from TG background, it seems very likely that the earlier identification of these proteins as regulators might have been artifacts of the random picking of colonies expressing or not expressing *agn43*. This clearly illustrates how ignoring phase variation of *agn43*, or its associated phenotypes, can introduce a strong bias into analyses of its expression in whole populations.

Finally, we investigated YfaL-mediated biofilm production. We found that Ag43-mediated aggregation can modulate the outcome of biofilm experiments designed to study other adhesins, and in particular experiments involving the popular microtiter plate assay. Although the mechanism by which Ag43 interferes with YfaL-mediated adhesion remains unclear, it is possible that the aggregation property of Ag43 is responsible for this interference. When growing biofilms in microtiter plates, large Ag43-mediated aggregates may be detached by washing procedures thereby reducing the number of attached bacteria and minimizing the effect of other adhesins. Our results are coherent with those observed previously for Ag43 interference with motility [50], thus suggesting that the presence/absence of Ag43 may modulate diverse cell surface structures. It is therefore possible that microtiter plate assays fail to identify all adhesins due to the random picking of Ag43+ ON populations interfering with the results. Inversely, care must be taken not to attribute autoaggregation properties to a protein without checking that it is not due to background expression of Ag43. Interestingly, physical interference by surface structures, including various pili, capsule or LPS, reciprocally affect Ag43 [51]–[53]. *E. coli* may have therefore evolved multiple mechanisms by which cell surface appendages can interfere or interact with each other, in a network at a higher order of regulation, overlapping with the known transcriptional regulatory network.

Our study clearly shows that careful assessment and monitoring the ON/OFF state of Ag43 in bacterial populations studied are required to avoid both misleading conclusions about *agn43* regulation, and misinterpretation of the adhesion and biofilm properties of other surface appendages.

## Supporting Information

Figure S1 **The quantity of ON cells in a wild-type (WT) culture determines its degree of aggregation.** Different amounts of a locked-ON (*ΔoxyR)* culture were mixed with a locked-OFF (*Δdam*) culture such that there were 0 to 100% ON cells; the mixtures were left to aggregate for 7 h at room temperature. **A.** Pictures of the settling cultures as with a WT ON (B) colony for reference, and the corresponding immunodetection using anti-Ag43 antibodies. **B.** Kinetics of aggregation of the same cultures. The degree of auto-aggregation is linearly correlated with the percentage of ON bacteria present in the culture. A threshold of ON bacteria (>25%) has to be reached before the auto-aggregation phenotype becomes visible and measurable. A WT (ON) colony, grown overnight in liquid LB medium, aggregates like a 75% ON culture, reflecting its natural mixed composition of Ag43+ and Ag43− cells.
(TIF)
