# Supplementary figures and images for: Did I Pick the Right Colony? Pitfalls in the Study of Regulation of the Phase Variable Antigen 43 Adhesin
Source: PLoS One. 2013 Sep 5;8(9):e73568. doi: 10.1371/journal.pone.0073568 (PMC3764049; doi:10.1371/journal.pone.0073568)

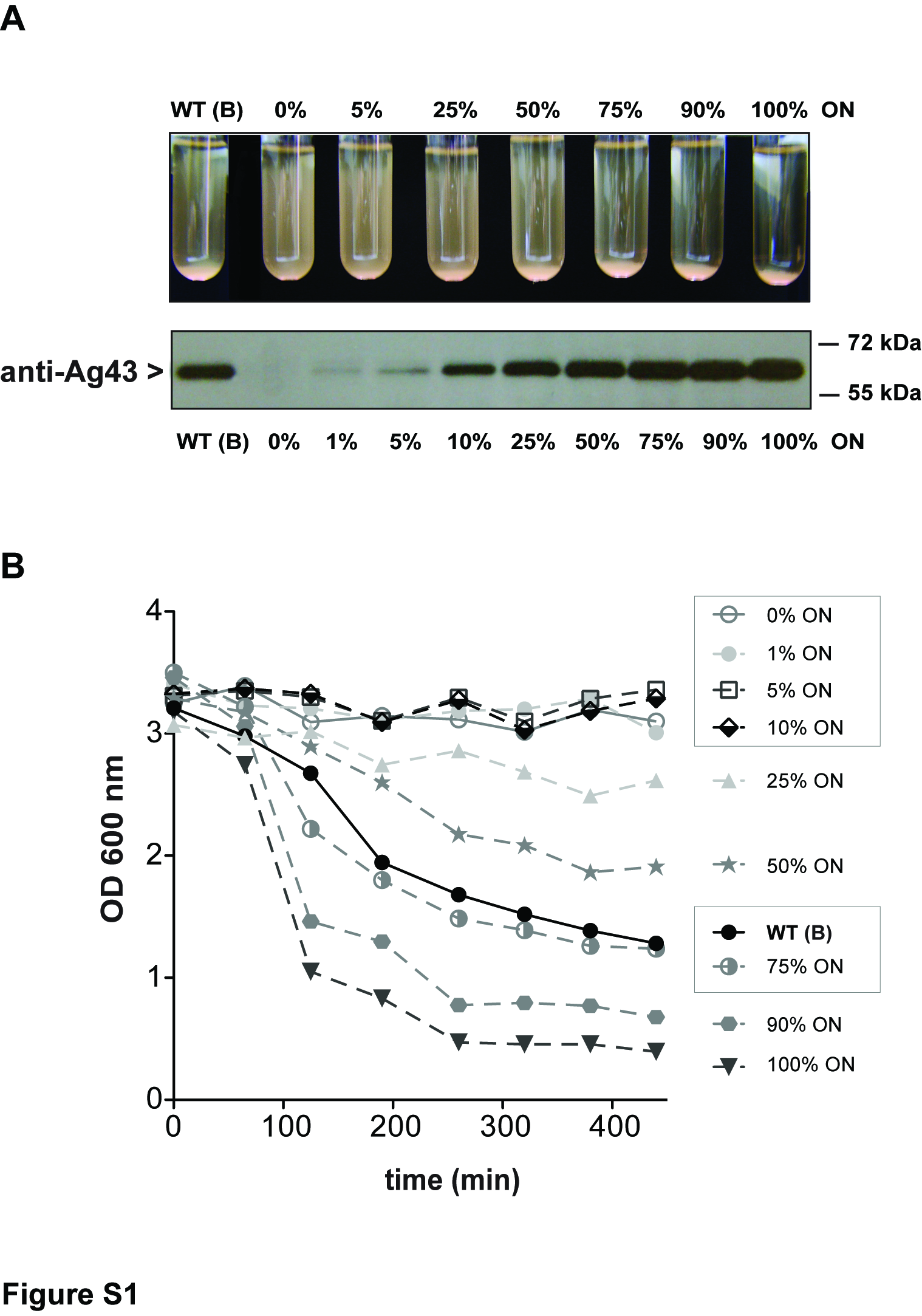

Supplement: Figure S1 — The quantity of ON cells in a wild-type (WT) culture determines its degree of aggregation. Different amounts of a locked-ON (ΔoxyR) culture were mixed with a locked-OFF (Δdam) culture such that there were 0 to 100% ON cells; the mixtures were left to aggregate for 7 h at room temperature. A. Pictures of the settling cultures as with a WT ON (B) colony for reference, and the corresponding immunodetection using anti-Ag43 antibodies. B. Kinetics of aggregation of the same cultures. The degree of auto-aggregation is linearly correlated with the percentage of ON bacteria present in the culture. A threshold of ON bacteria (>25%) has to be reached before the auto-aggregation phenotype becomes visible and measurable. A WT (ON) colony, grown overnight in liquid LB medium, aggregates like a 75% ON culture, reflecting its natural mixed composition of Ag43+ and Ag43− cells. (TIF) [file pone.0073568.s001.tif]
